# Editorial Expression of Concern: Loss of *Smad4* promotes aggressive lung cancer metastasis by de-repression of PAK3 via miRNA regulation

**DOI:** 10.1038/s41467-026-71023-9

**Published:** 2026-03-25

**Authors:** Xiaohong Tan, Lu Tong, Lin Li, Jinjin Xu, Shaofang Xie, Lei Ji, Junjiang Fu, Qingwu Liu, Shihui Shen, Yun Liu, Yanhui Xiao, Feiran Gao, Robb E. Moses, Nabeel Bardeesy, Yanxiao Wang, Jishuai Zhang, Longying Tang, Lei Li, Kwok-kin Wong, Dianwen Song, Xiao Yang, Jian Liu, Xiaotao Li

**Affiliations:** 1 State Key Laboratory of Proteomics, Beijing Proteome Research Center, National Center for Protein Sciences (Beijing), Beijing Institute of LifeOmics, Beijing, China; 2 Institute of Biomedical Engineering, School of Medical Instrument and Food Engineering, University of Shanghai for Science and Technology, Shanghai, China; 3 Shanghai Key Laboratory of Regulatory Biology, Institute of Biomedical Sciences, School of Life Sciences, East China Normal University, Shanghai, China; 4 Department of Orthopedic Oncology, Changzheng Hospital, The Second Military Medical University, Shanghai, China; 5 Key Laboratory of Epigenetics and Oncology, The Research Center for Preclinical Medicine, Southwest Medical University, Luzhou, Sichuan China; 6 Zhejiang University-University of Edinburgh Institute (ZJU-UoE Institute), Zhejiang University School of Medicine, International Campus, Zhejiang University, Haining, China; 7 Department of Respiratory and Critical Care Medicine, the Second Affiliated Hospital, Zhejiang University School of Medicine, Zhejiang University, Hangzhou, China; 8 Department of Molecular and Cellular Biology, Dan L. Duncan Cancer Center, Baylor College of Medicine, One Baylor Plaza, Houston, TX USA; 9 Cancer Center, Massachusetts General Hospital, Boston, MA USA; 10 Shanghai Changning Maternity and Infant Health Hospital, Shanghai, China; 11 Division of Hematology & Medical Oncology, Laura and Isaac Perlmutter Cancer Center, New York University Langone Medical Center, New York, NY USA; 12 Department of Orthopedics, Shanghai General Hospital, Shanghai Jiao Tong University, School of Medicine, Shanghai, China

Editorial Expression of Concern to: *Nature Communications* 10.1038/s41467-021-24898-9, published online 11 August 2021

The Editors would like to alert the readers that concerns have been raised regarding similarities between different images in this article. Specifically,

- the *0 h, SPK-1 assay* in Figure 2c appears to overlap with the *0 h, SPK Antagonist miR-543* assay in Figure 2 g;
- the *p-JNK* and *p-Jun* panels in the top row of Figure 6 h appear to overlap partially.

The Authors provided some source data on request from the Editors, but this was insufficient to address the concerns raised. Readers are advised to interpret these results with caution.

Xiaotao Li, Lu Tong, Lei Li, and Kwok-kin Wong agree with this Editorial Expression of Concern. Jian Liu did not explicitly state whether or not they agree with this Editorial Expression of Concern. Xiaohong Tan, Lin Li, Jinjin Xu, Shaofang Xie, Lei Ji, Junjiang Fu, Qingwu Liu, Shihui Shen, Yun Liu, Yanhui Xiao, Feiran Gao, Robb E. Moses, Nabeel Bardeesy, Yanxiao Wang, Jishuai Zhang, Longying Tang, Dianwen Song, and Xiao Yang did not respond to correspondence about this Editorial Expression of Concern.

