# Supplementary material for: Loss of Smad4 promotes aggressive lung cancer metastasis by de-repression of PAK3 via miRNA regulation
Source: Nat Commun. 2021 Aug 11;12:4853. doi: 10.1038/s41467-021-24898-9 (PMC8357888; doi:10.1038/s41467-021-24898-9)
Supplement: Supplementary file 1 — Supplementary Information [file 41467_2021_24898_MOESM1_ESM.pdf]

The title page for the supplementary information file of the manuscript entitled "Loss of Smad4 promotes aggressive lung cancer metastasis by de-repression of PAK3 via miRNA regulation"

Supplementary Fig. 1. Smad4 deletion accelerates lung tumorigenesis and metastasis.

Supplementary Fig. 2. Abrogation of Smad4 promotes the lung cancer cell migration and invasion.

Supplementary Fig. 3. PAK3 is a downstream effector of Smad4 mediating lung cancer cell metastasis.

Supplementary Fig. 4. PAK3 enhances the JNK-Jun signal pathway.

Supplementary Fig. 5. Smad4 negatively regulates PAK3 via transactivation of miR-495 and miR-543 expression.

Supplementary Fig. 6. miR-495 and miR-543 were direct targets of Smad4 activation.

Supplementary Fig. 7. MiR-495 and miR-543 directly bind to the PAK3, UTR and attenuate metastatic potential of lung cancer cells *in vitro* and *in vivo*.

Supplementary Fig. 8. RAS (G12D) and P53 high expressed in human lung cancer metastatic tissues.

Supplementary Fig. 9. The expression of Smad4 and PAK3/JNK/Jun/RAS (G12D)/P53 in human early/advanced lung cancer tissues.

Supplementary Fig. 10. Pearson correlation analysis of Fig.7A-B.

Supplementary Fig. 11. Full scan images for the figures 2a, 2d, 3f, 4a-b, 6b-c, 6i.

Supplementary Fig. 12. Full scan images for the supplementary figures 2c, 2g, 3a, 4a-c, 4i, 5a, 5c, 6a, 6d, 7a, 7c.

Sup. Table 1. Sequence of primers used in the manuscript.

Supplementary Fig. 1

a

Genotype	Frequency of metastasis	Site of metastasis
Kras^{G12D}	0/57 (0%)	
Smad4$^{fl/fl}$; *Kras*^{G12D} (SK)	3/54 (5.6%)	3/54 to pleura<br>2/54 to lymph node
p53$^{fl/fl}$; *Kras*^{G12D} (PK)	6/44 (13.6%)	5/44 to lymph node<br>2/44 to thymus<br>1/44 to heart
Smad4$^{fl/fl}$; *p53*$^{fl/fl}$;*Kras*^{G12D} (SPK)	43/84 (51.2%)	37/84 to lymph node<br>23/84 to thymus<br>22/84 to kidney<br>10/84 to pleura<br>8/84 to heart<br>5/84 to bone<br>4/84 to liver<br>3/84 to muscle

b

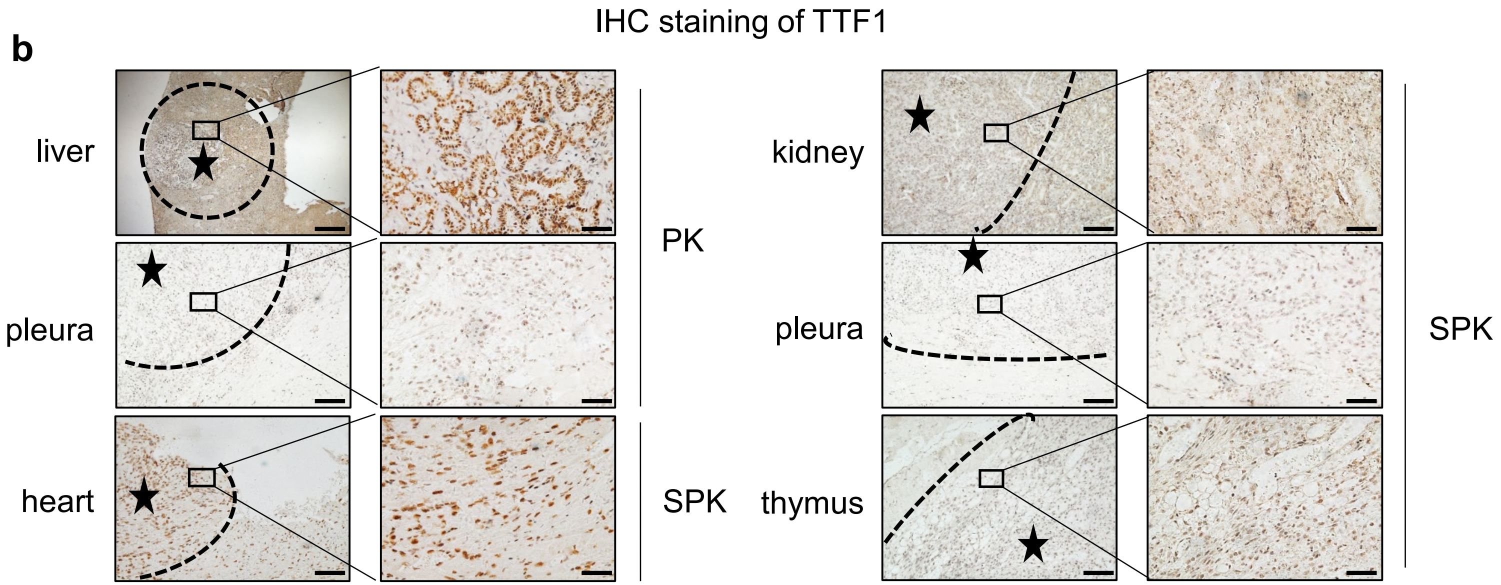

c

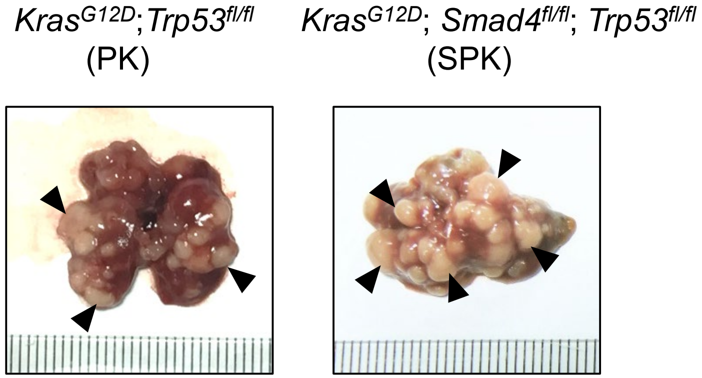

d

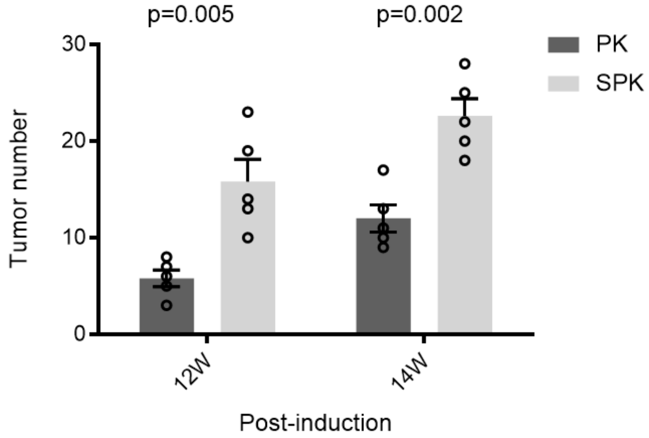

e

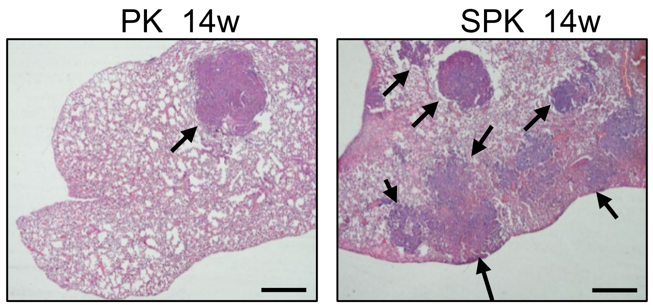

f

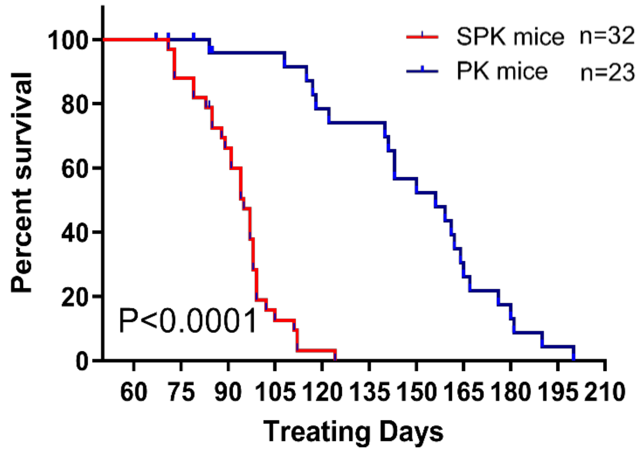

Supplementary Fig. 1 . Smad4 deletion accelerates lung tumorigenesis and metastasis

a. Statistical analyses of lung cancer metastases frequency and the site of metastasis in *Kras*^{G12D} (n=57), *Smad4*$^{fl/fl}$*; Kras*^{G12D} (n=54), *p53*$^{fl/fl}$*; Kras*^{G12D} (n=44), *Smad4*$^{fl/fl}$*; p53*$^{fl/fl}$*; Kras*^{G12D} (n=84) mice.

b. Representative expression of TTF1 marker in PK and SPK adenocarcinoma and metastatic tumor tissues, including liver, pleura from PK mouse and heart, kidney pleura, thymus from SPK mouse. Similar staining results were observed in a group of mice, whose information listed in Sup. Data 5. Asterisk indicates the area of metastatic tumors. Scale bar, 100 μm (magnification, ×10), 25 μm (magnification, ×40).

c-e. Representative pictures, H&E staining results and statistical analyses of lung tumors of PK or SPK mouse. PK and SPK mouse took the same amount of time to treat with Ad-Cre. . Scale bar, 100 μm (magnification, ×10). P<0.01; Data presented are the mean ± SEM from three biologically independent samples (n = 3); as determined by two-tailed Student's *t* test.

f. Kaplan-Meier curves with two-sided log-rank test to analyze the survival statistics of PK and SPK mice with lung cancer. n=23 in PK and n=32 in SPK mouse groups. P = 6.52332E-07. The survival range of all the mouse was between 10 and 28 weeks after the mouse infected with adeno-Cre.

Supplementary Fig. 2

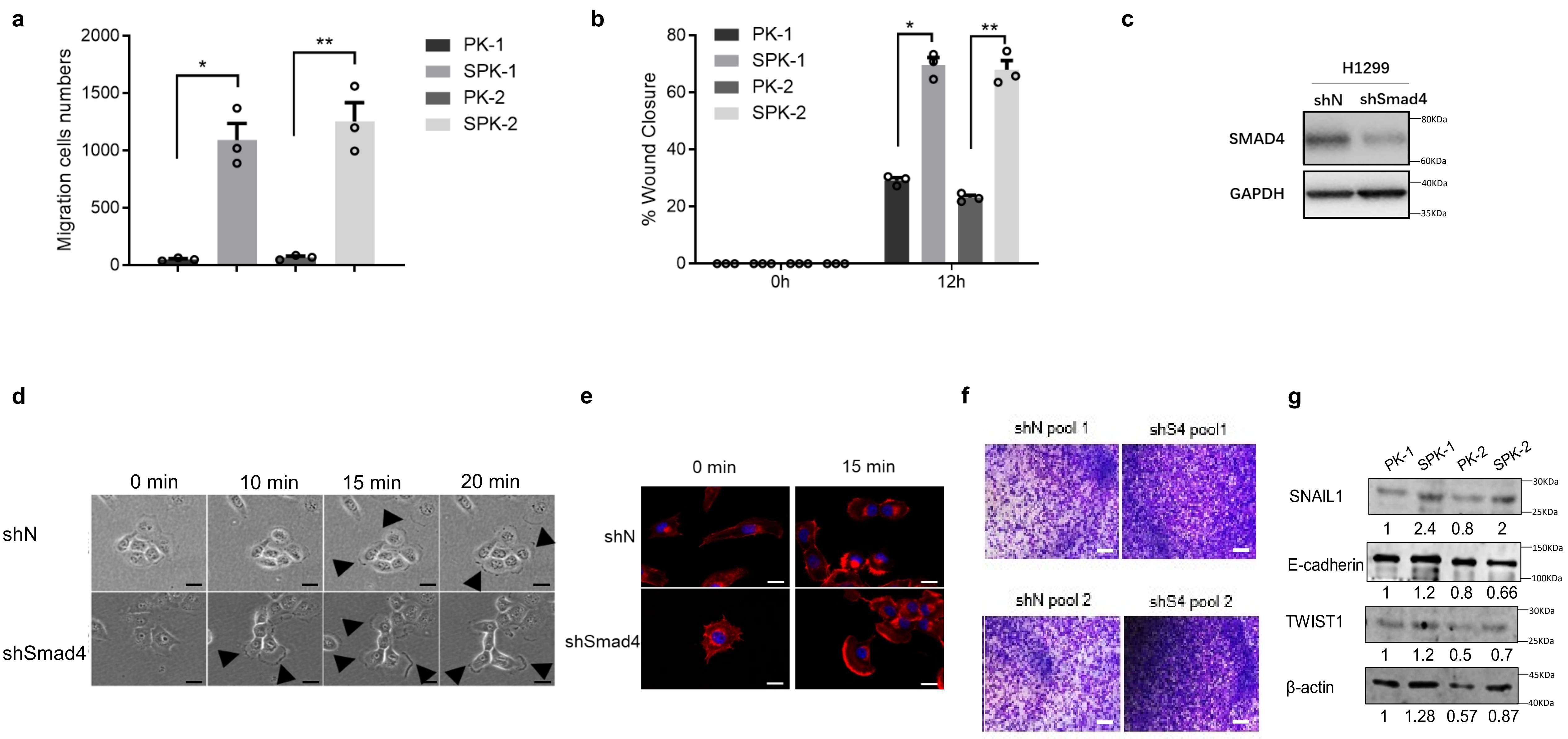

Supplementary Fig. 2. Abrogation of Smad4 promotes the lung cancer cell migration and invasion

a. Statistics of PK1/2 and SPK1/2 migration cells number of transwell assay(Figure 2B). Data presented are the mean ± SEM from three biologically independent samples (n = 3); *, p = 0.001914887; **, p = 0.002024024; as determined by two-tailed Student's t test. **b.** Wound healing percentage of PK1/2 and SPK1/2 of Figure 2C was quantified. Data presented are the mean ± SEM from three biologically independent samples (n = 3); *, p = 0.000128336; **, p = 0.000199573; as determined by two-tailed Student's t test. **c.** Detection of the expression of Smad4 in H1299 shN and H1299 shSmad4 cells, and the experiment was repeated three times independently with similar results. **d.** H1299 shN and H1299 shSmad4 cells were serum-starved 24 h, then stimulated with serum in Super-resolution Multiphoton Confocal Microscope and taken pictures each 30 sed. The black triangles refer to the pseudopodium, and the experiment was repeated three times independently with similar results. Scale bar, 25 μm. **e.** H1299 shN and H1299 shS4 cells were serum-starved 24 h before stimulation with serum for 0min and 15min at 37 ° C. Cells were then fixed and stained with Rhodamine-Phalloidin (Red, F-actin) and DAPI (Blue, nucleus). The speed of cytoskeleton reconstruction, the number and length of cilia and pseudopodium, the change of cell morphology all represent the cell migration activity. The white triangles indicate cilia and pseudopodium. Scale bar, 25 μm (magnification, ×40) , and the experiment was repeated three times independently with similar results. **f.** Invasion chamber analysis of H1299 cells, stained by 0.5% crystal violet. ShN: shRNA for control; shS4: shRNA for *SMAD4*, and the experiment was repeated three times independently with similar results. Scale bar, 100 μm. **g.** Western blot analysis of protein expression in PK and SPK cells, and the experiment was repeated three times independently with similar results.

Supplementary Fig. 3

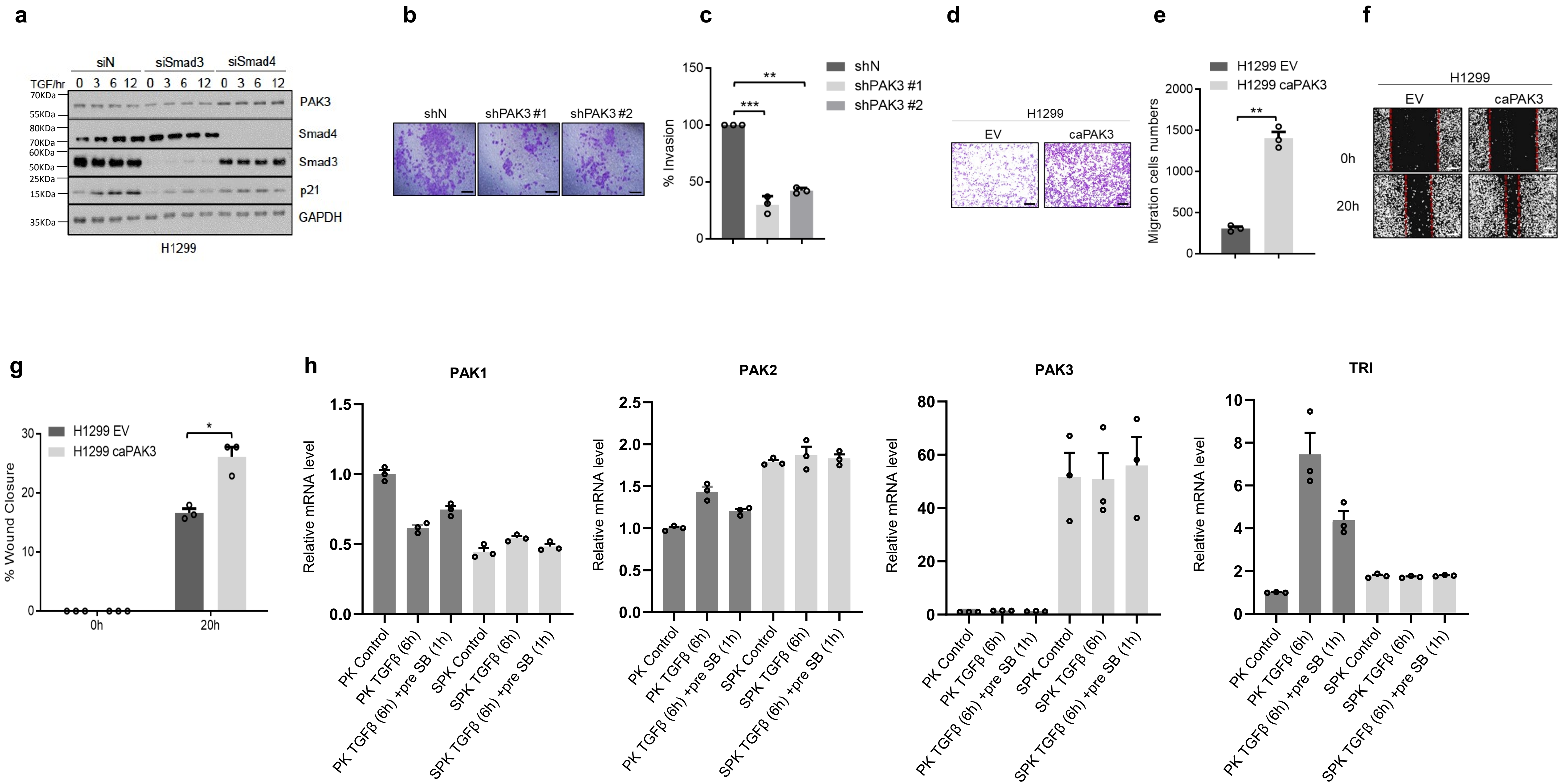

Supplementary Fig. 3. PAK3 is a downstream effector of Smad4 mediating lung cancer cell metastasis

a. Expression of PAK3, Smad3 and Smad4 when silencing Smad3 or Smad4 with the treatment of TGFβ in H1299 cells, and the experiment was repeated three times independently with similar results.

b. The migration ability of SPK shN and shPAK3(#1/#2) cells was tested by Transwell assay. Scale bar, 100 μm.

c. The percentage of invasive cells of Transwell assay. Data presented are the mean ± SD from three biologically independent samples (n = 3); **, p = 2.4079E-06; ***, p = 8.7927E-05; as determined by two-tailed Student's t test.

d. Over-expressed PAK3 in H1299 cells by adeno-associated virus (H1299-caPAK3). The migration ability of H1299-Vector and H1299-caPAK3 cells was compered by Transwell assay. Scale bar, 100 μm.

e. Statistical analyses of invasion cells. Data presented are the mean ± SEM from three biologically independent samples (n = 3); **, p = 0.000145633; as determined by two-tailed Student's t test.

f. The migration ability of H1299-Vector and H1299-caPAK3 cells was compered by wound healing assay. Scale bar, 100 μm.

g. Statistical analyses of wound closure percentage. Data presented are the mean ± SEM from three biologically independent samples (n = 3); *, p = 0.005867988; as determined by two-tailed Student's t test.

h. qRT-PCR analysis of gene expression in PK and SPK cells. Data presented are the mean ± SEM from three biologically independent samples (n = 3).

Supplementary Fig. 4

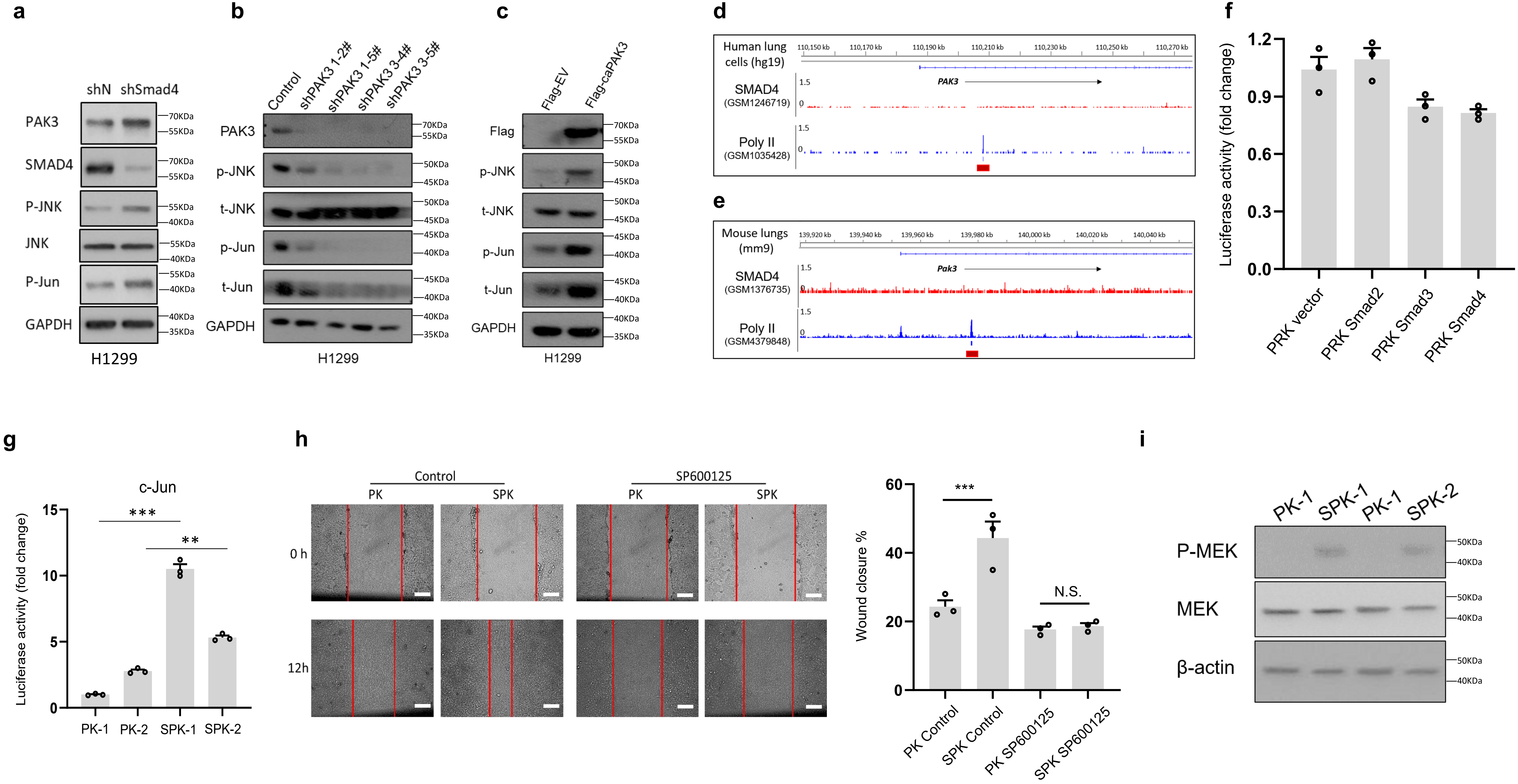

Supplementary Fig. 4. PAK3 enhances the JNK-Jun signal pathway

a. Expression of PAK3, JNK-Jun related factors in H1299 shN and H1299 shSmad4 cells, , and the experiment was repeated three times independently with similar results.

b. Expression of PAK3, JNK-Jun related factors in H1299 and several H1299 shPAK3 (inhibited expression of PAK3) cells, and the experiment was repeated three times independently with similar results.

c. Expression of PAK3, JNK-Jun related factors in H1299 and H1299 Flag-caPAK3 cells, and the experiment was repeated three times independently with similar results.

d-e. Analysis of the SMAD4 ChIP-Seq in human (GSM1246719) and mouse (GSM1376735) lungs in the published database.

f. Luciferase assay on mouse Pak3 Promoter. Data represent means $\pm$ SEM.

g. RT-qPCR analysis of gene expression in PK and SPK cells. Data presented are the mean $\pm$ SEM from three biologically independent samples ($n = 3$); ***, $p = 1.35682E-05$; **, $p = 0.000188084$; as determined by Student's t test.

h. Migration assay of PK and SPK cells. Data presented are the mean $\pm$ SEM from three biologically independent samples ($n = 3$); ***, $p = 0.017824971$; as determined by Student's t test. Scale bar, 100 μm.

i. Western blot analysis of protein expression in PK and SPK cells, and the experiment was repeated three times independently with similar results.

Supplementary Fig. 5

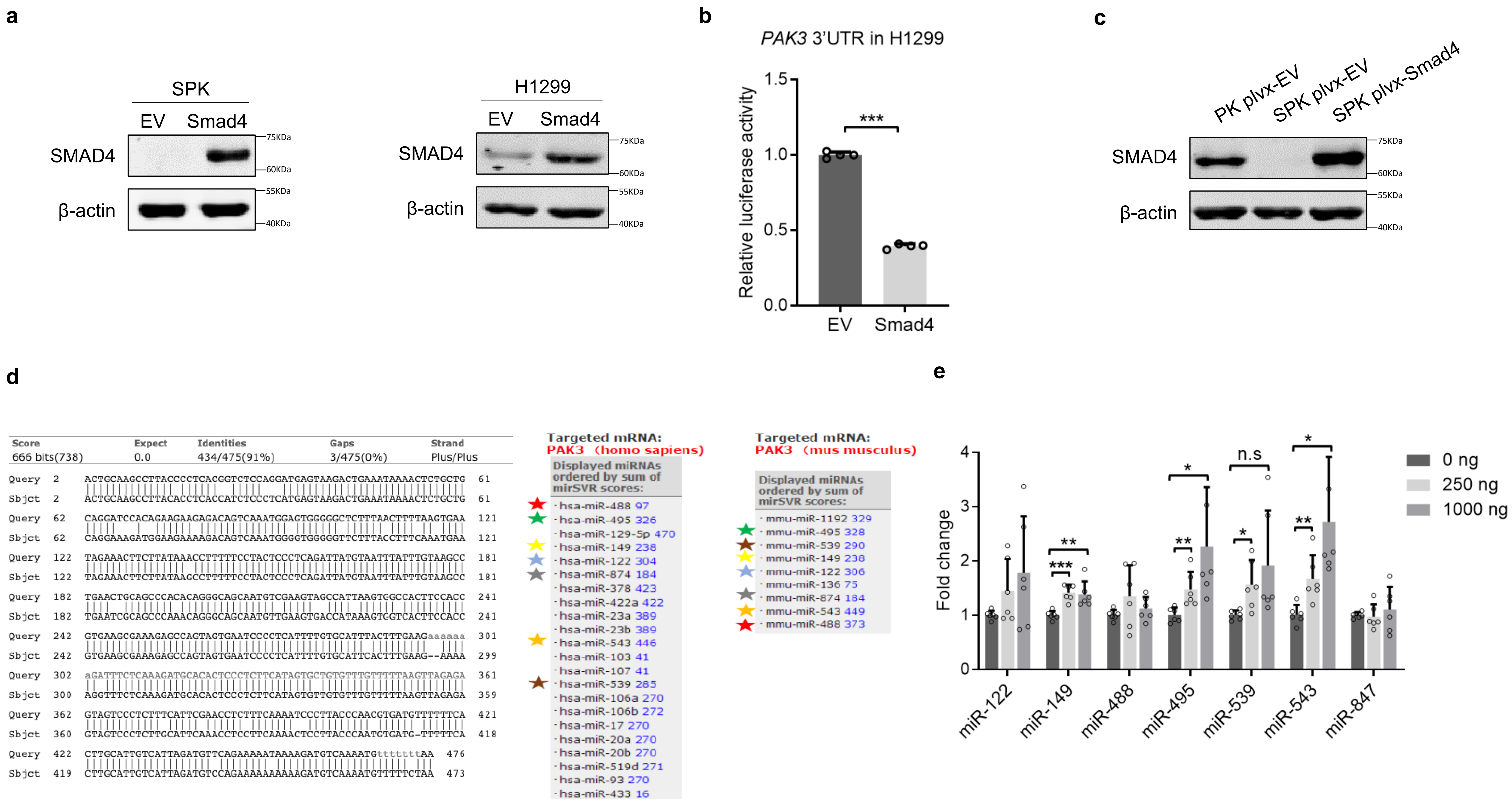

Supplementary Fig. 5. Smad4 negatively regulates PAK3 via transactivation of miR-495 and miR-543 expression

a. The expression of SMAD4 in normal and overexpressing SMAD4 SPK cells was detected by western blotting assay, and the experiment was repeated three times independently with similar results.

b. Luciferase reporter assay showed that overexpression of Smad4 decreased PAK3 expression level in H1299 cells. The expression of SMAD4 in normal and overexpressing SMAD4 H1299 cells was detected by western blotting assay. Data presented are the mean ± SEM from three biologically independent samples (n = 3); ***, p =6.40919E-09; as determined by Student's t test.

c. The expression of SMAD4 in normal PK cells, normal and overexpressing SMAD4 SPK cells was detected by western blotting assay, and the experiment was repeated three times independently with similar results.

d. The homology of the 3'UTR regions of murine and human PAK3 (left panel) and several PAK3 highly correlated miRNAs were evaluated (right panel).

e. RT-qPCR analysis of three microRNAs, miR-495, miR-539 and miR-543 positively regulated by Smad4 in a dose-dependent manner in H1299 cells. Data presented are the mean ± SEM from three biologically independent samples (n = 3); *** (miR-149), p = 0.000124246; ** (miR-149), p = 0.004513014; ** (miR-495), p = 0.008839299; * (miR-495), p = 0.018641575; * (miR-539), p = 0.01343452; ** (miR-543), p = 0.00630325; * (miR-543), p = 0.006080816; as determined by Student's t test.

Supplementary Fig. 6

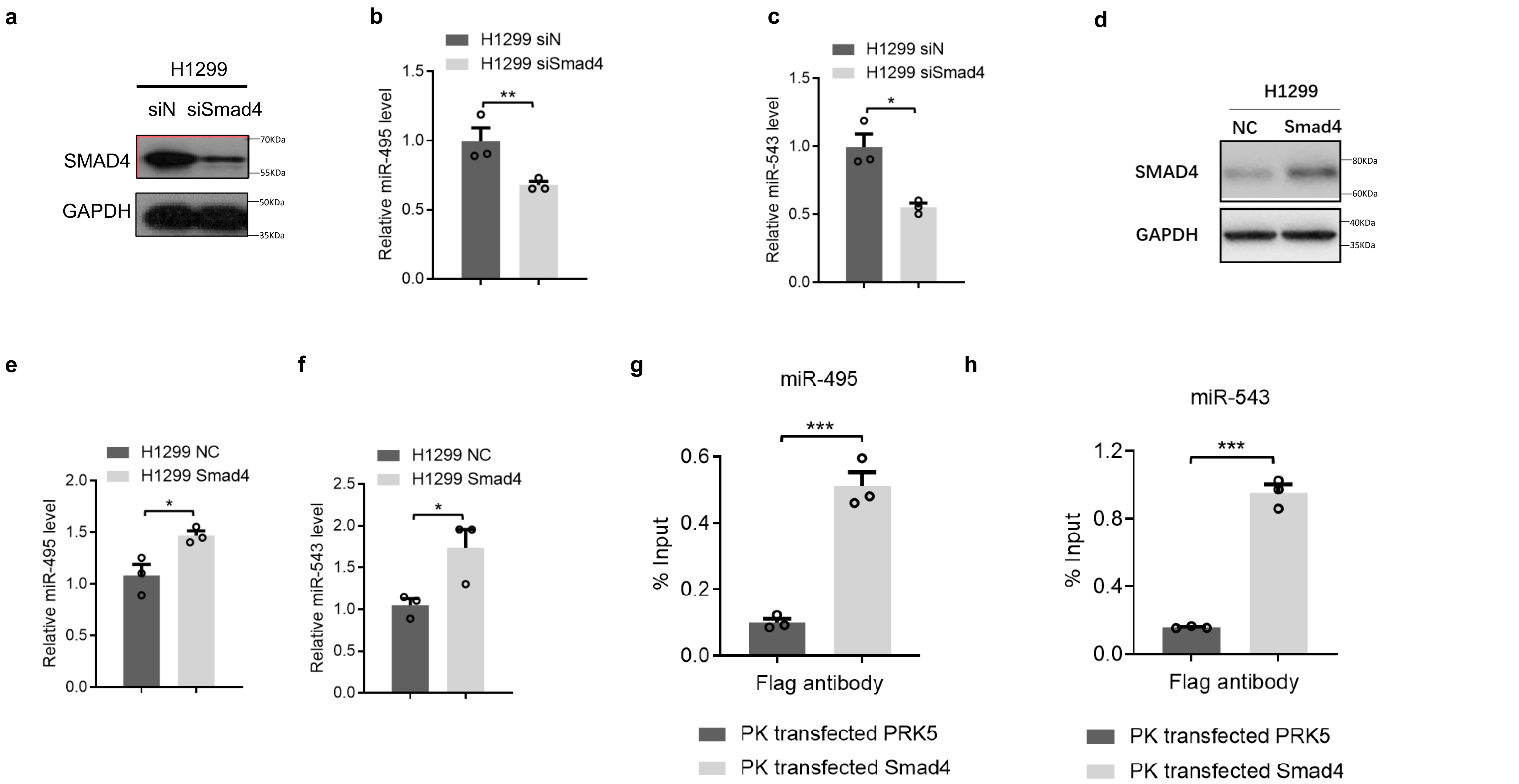

Supplementary Fig. 6. miR-495 and miR-543 were direct targets of Smad4 activation.

a. The expression of SMAD4 in H1299 shN and H1299 shSmad4 cells was detected by western blotting assay.

b-c. The RNA expression of miR-495 and miR-543 were detected in H1299 shN and H1299 shSmad4 cells. Data presented are the mean ± SEM from three biologically independent samples (n = 3); *, p = 0.012203029; **, p = 0.034969898； as determined by two-tailed Student’s t test.

d. The expression of SMAD4 in normal and overexpressing SMAD4 H1299 cells was detected by western blotting assay.

e-f. The RNA expression of miR-495 and miR-543 were detected in normal and overexpressing SMAD4 H1299 cells. Data presented are the mean ± SEM from three biologically independent samples (n = 3); * (e), p = 0.028324677; * (f), p = 0.040769922; as determined by two-tailed Student’s t test.

g-h. The interaction between Smad4 and miR-543 in SPK cells were verified by ChIP assay. Data presented are the mean ± SEM from three biologically independent samples (n = 3); *** (g), p = 0.000687613; *** (h), p = 8.79594E-05; as determined by two-tailed Student’s t test.

Supplementary Fig. 7

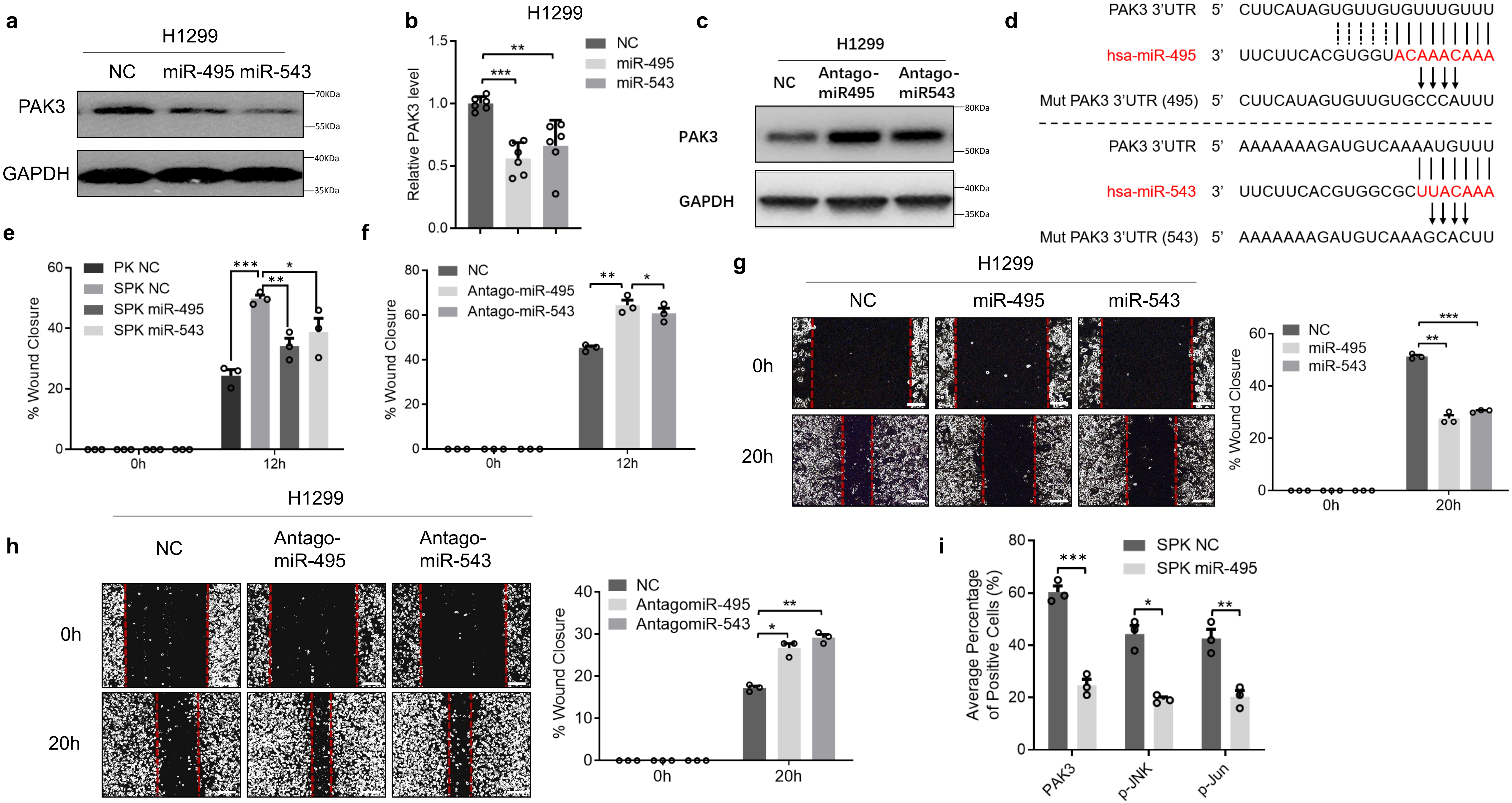

Supplementary Fig. 7. MiR-495 and miR-543 directly bind to the PAK3, UTR and attenuate metastatic potential of lung cancer cells *in vitro* and *in vivo*

a-b. The protein and mRNA expression level of PAK3 was tested by western blotting and qPCR in H1299 NC, H1299 miR-495 and H1299 miR-543 cells. Data presented are the mean ± SEM from three biologically independent samples (n = 3); **, p = 0.002826925; ***, p = 1.5821E-05 ; as determined by two-tailed Student's t test.

c. The protein expression level of PAK3 was detected by western blotting in H1299 NC, H1299 antagomiR-495 and H1299 antagomiR-543 cells, and the experiment was repeated three times independently with similar results.

d. The sketch map of PAK3 3'UTR mutant (miR-495/miR-543) sequence.

e-f. Statistical analyses of wound closure percentage in Figure 6F and 6G. Data presented are the mean ± SEM from three biologically independent samples (n = 3); *** (e), p = 0.000394471; ** (e), p = 0.005596008; * (e), p = 0.049110999; ** (f), p = 0.001446721; * (f), p = 0.003270911; as determined by two-tailed Student's t test.

g-h. The migration ability was verified by wound healing assay in H1299 cells which were treated with miR-495/543 or antogomiR-495/543 separately (left). Statistical analyses of wound closure percentage (right). Data represent means ± SEM; ** (g), p = 7.15495E-05; *** (g), p = 4.92185E-06; ** (h), p = 0.0001826; * (h), p = 0.001339582; as determined by two-tailed Student's t test. Scale bar, 100 μm.

i. Statistics of positive cells average percentage of Fig.6H. Data presented are the mean ± SEM from three biologically independent samples (n = 3); *** (e), p = 0.000440696; ** (e), p = 0.005964323; * (e), p = 0.001820757; as determined by two-tailed Student's t test.

Supplementary Fig. 8

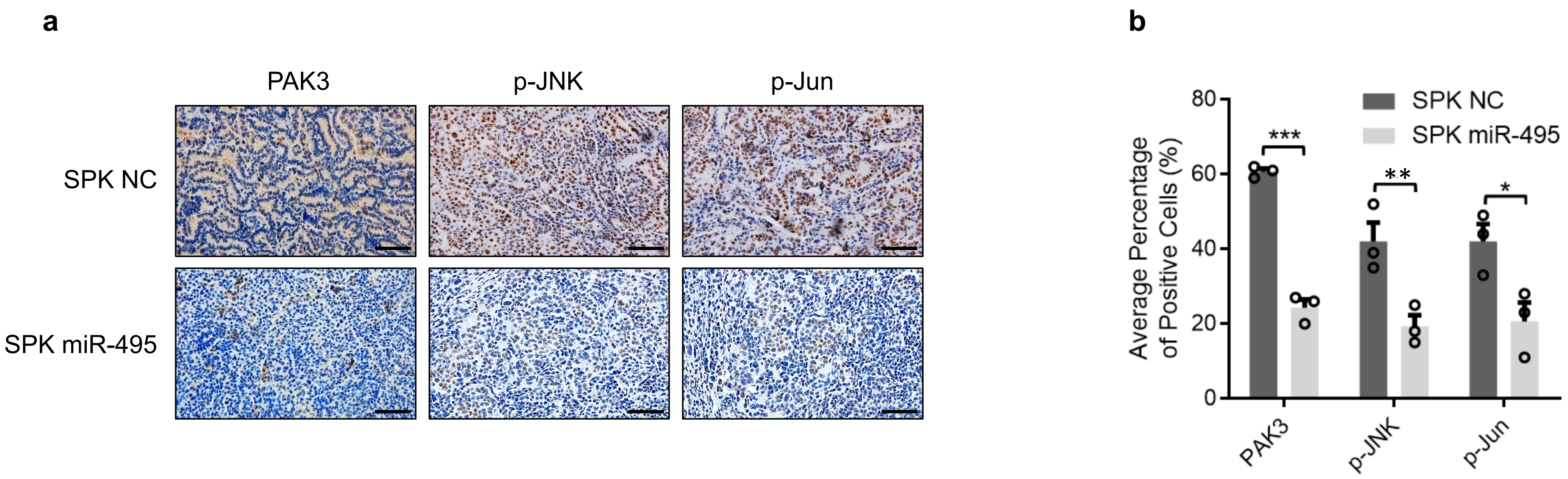

Supplementary Fig. 8. RAS (G12D) and P53 high expressed in human lung cancer metastatic tissues.

a. The expression level of PAK3, p-JNK and p-Jun of the other parts of SPK NC and miR-495 mouse lung cancer tumors were detected by IHC staining. Scale bar, 25 μm (magnification, ×40).

b. Statistics of positive cells average percentage of (A). Data presented are the mean ± SEM from three biologically independent samples (n = 3); ***, p = 0.000103347; **, p = 0.018693204; *, p = 0.036704115; as determined by two-tailed Student's t test.

Supplementary Fig. 9

a

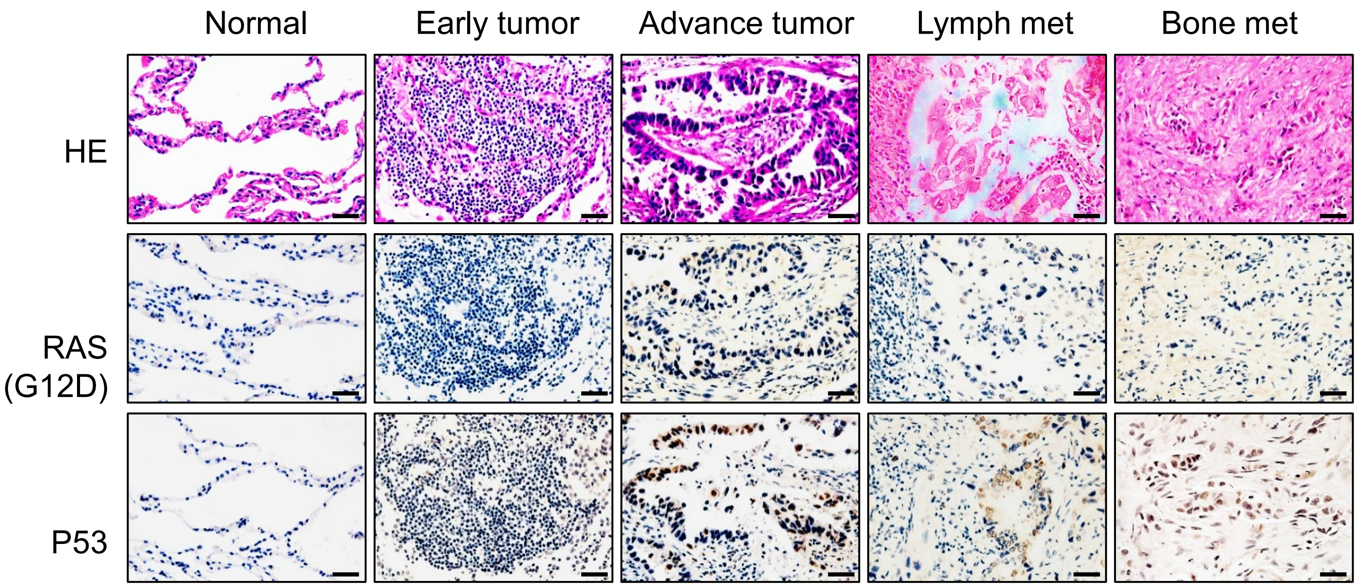

b

Protein level	Samples	-	+	++	+++	Total samples
RAS (G12D)	Normal	15 (100%)	0 (0.0%)	0 (0.0%)	0 (0.0%)	15
	Early tumor	14 (93.3%)	1 (6.7%)	0 (0.0%)	0 (0.0%)	15
	Advanced tumor	11 (91.7%)	0 (4.5%)	0 (0.0%)	1 (8.3%)	12
	Lymph met	20 (90.9%)	1 (4.5%)	1 (4.5%)	0 (0.0%)	22
	Bone met	7 (87.5%)	0 (0.0%)	1 (12.5%)	0 (0.0%)	8
P53	Normal	11 (73.3%)	3 (20.0%)	1 (6.7%)	0 (0.0%)	15
	Early tumor	2 (13.3%)	5 (33.3%)	6 (40.0%)	2 (13.3%)	15
	Advanced tumor	0 (0.0%)	2 (16.7%)	7 (58.3%)	3 (25.0%)	12
	Lymph met	0 (0.0%)	4 (18.2%)	9 (40.9%)	9 (40.9%)	22
	Bone met	1 (12.5%)	2 (25.0%)	4 (50.0%)	1 (12.5%)	8

c

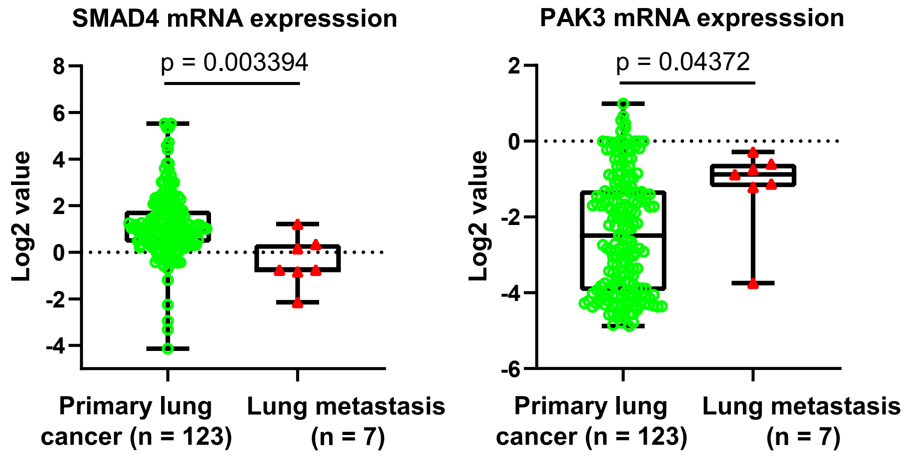

d

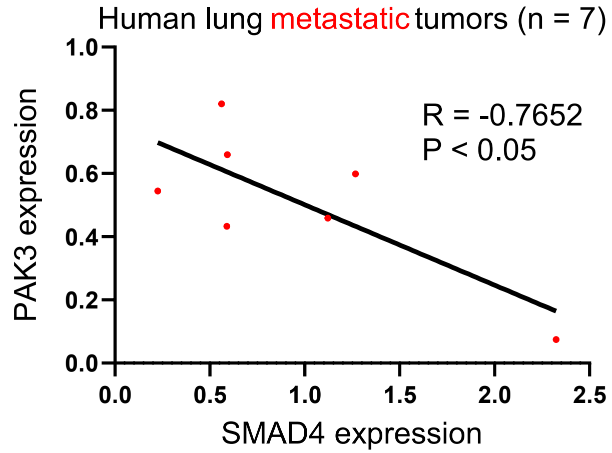

Supplementary Fig. 9. The expression of Smad4 and PAK3/JNK/Jun/RAS (G12D)/P53 in human early/advanced lung cancer tissues.

a. Representative HE and IHC images of RAS (G12D)/P53 in human normal lung tissues, early/advanced lung cancer tissues and lung cancer lymph/bone metastatic tumor tissues. Scale bar, 25 μm (magnification, ×40).

b. Statistics of positively stained percentages in human metastatic lung cancer and normal lung tissues (n=15); early tumor (n=15); advanced tumor (n=12); lymph nodes metastases (n=22); bone metastases (n=8).

c. The gene expression information of 123/7 cases of lung cancer patients primary/metastasis tumor tissues from the raw data of a published paper (PMID: 11707567) were collected and analyzed the expression of SMAD4 and PAK3 using Box & whiskers (largest value to smallest value). Two-tailed Student's t test was conducted.

d. Pearson correlation analysis of expression between PAK3 and SMAD4 in human lung cancer metastatic tumors(n=7). P = 0.045008; as determined by two-tailed Student's t test.

Supplementary Fig. 10

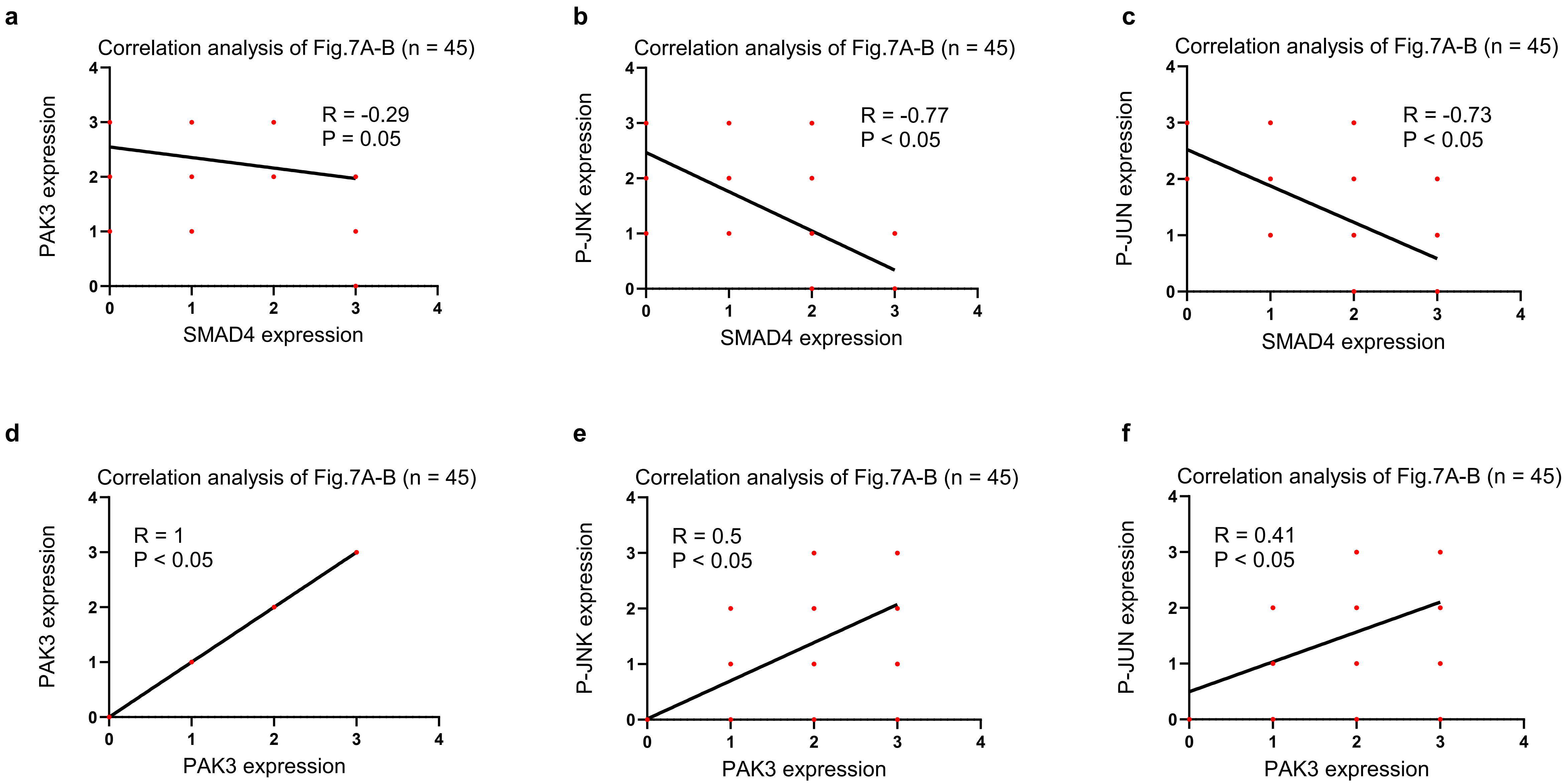

Supplementary Fig. 10. Pearson correlation analysis of Fig.7A-B.

a. Pearson correlation analysis of expression between PAK3 and SMAD4 in human samples in Fig.7A-B. P = 0.054476; as determined by two-tailed Student's t test.

b. Pearson correlation analysis of expression between p-JNK and SMAD4 in human samples in Fig.7A-B. P = 5.265039e-10; as determined by two-tailed Student's t test.

c. Pearson correlation analysis of expression between P-JUN and SMAD4 in human samples in Fig.7A-B. P = 9.764420e-09; as determined by two-tailed Student's t test.

d. Pearson correlation analysis of expression between PAK3 and PAK3 in human samples in Fig.7A-B. P = 0.000000e+00; as determined by two-tailed Student's t test.

e. Pearson correlation analysis of expression between PAK3 and P-JNK in human samples in Fig.7A-B. P = 0.0005; as determined by two-tailed Student's t test.

f. Pearson correlation analysis of expression between PAK3 and P-JUN in human samples in Fig.7A-B. P = 0.005762; as determined by two-tailed Student's t test.

Supplementary Fig. 11. Full scan images for the figures 2a, 2d, 3f, 4a-b, 6b-c, 6i

Figure 2a – SMAD4

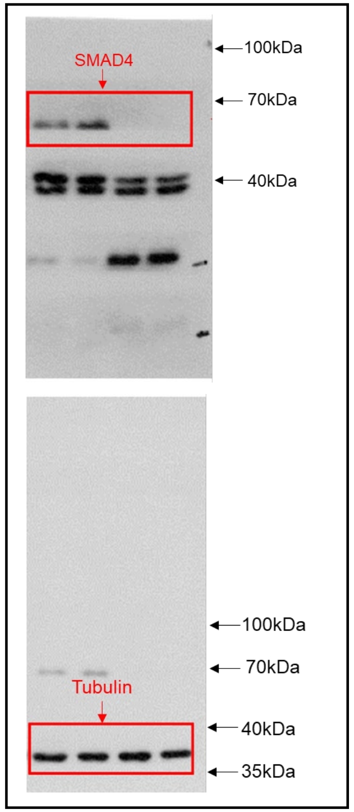

Figure 2d – SMAD4 GAPDH

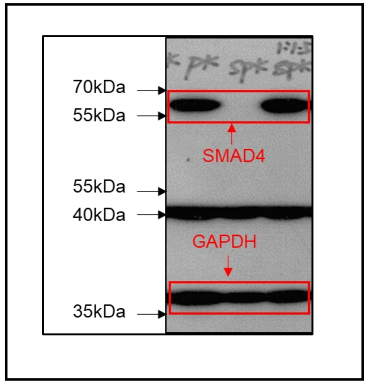

Figure 3f – PAK3 / Tubulin

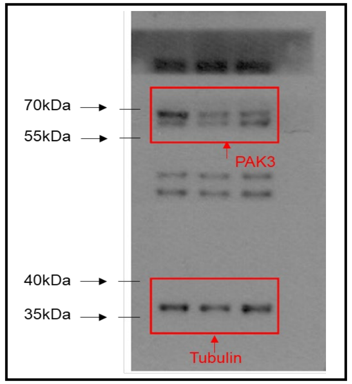

Figure 4a – SMAD4 PAK3 P-JNK T-JNK P-JUN T-JUN β-ACTIN

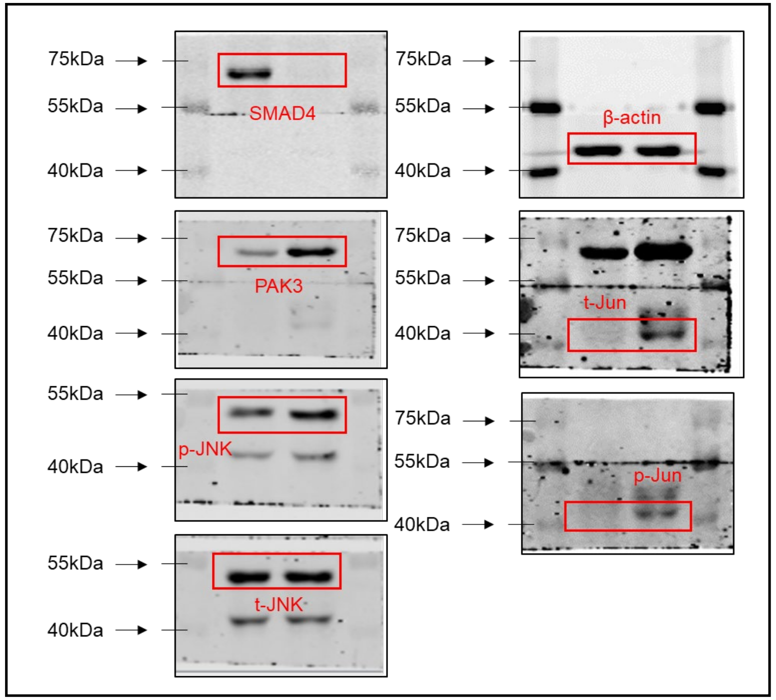

Figure 4b – SMAD4 PAK3 P-JNK T-JNK P-JUN T-JUN β-ACTIN

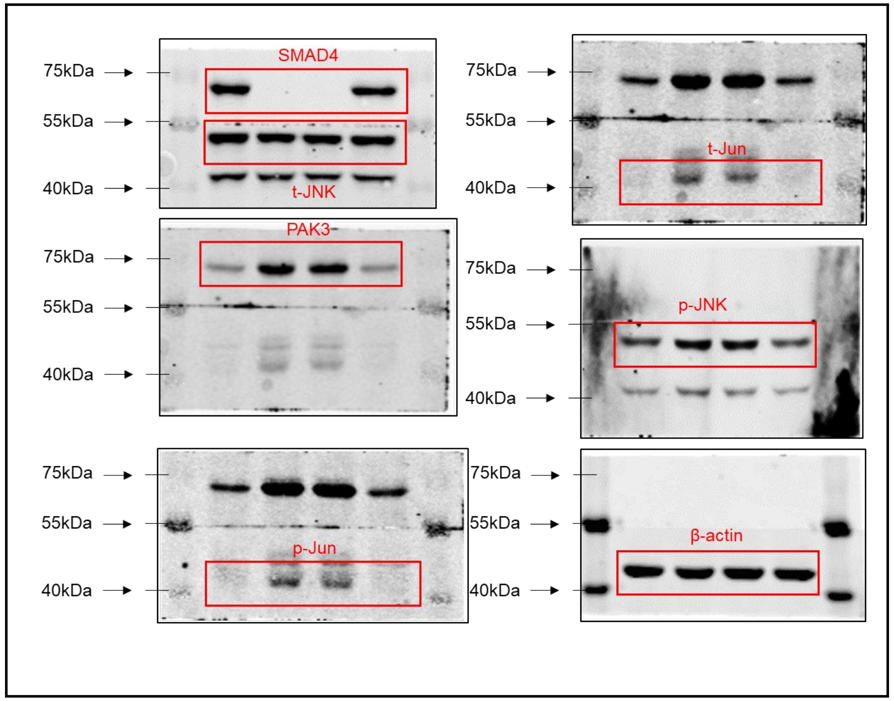

Figure 6b – PAK3 GAPDH

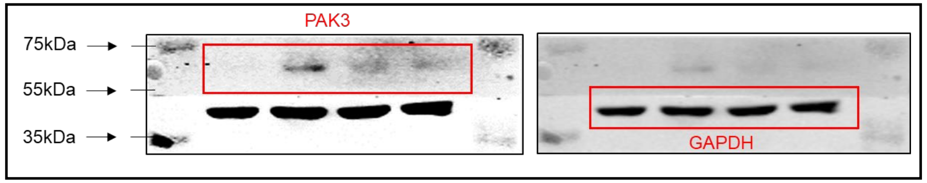

Figure 6c – PAK3 GAPDH

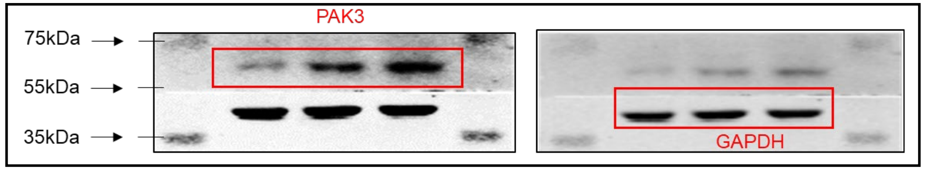

Figure 6i – PAK3 p-JNK P-JUN GAPDH

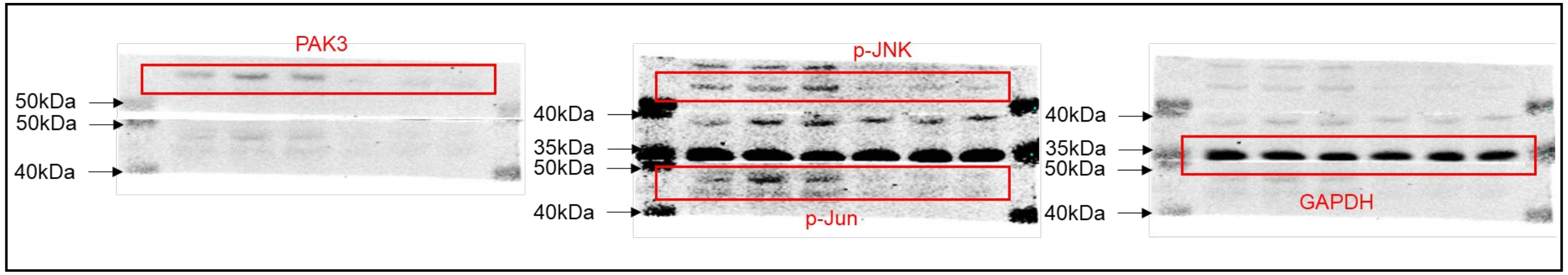

Supplementary Fig. 12. Full scan images for the supplementary figures 2c, 2g, 3a, 4a-c, 4i, 5a, 5c, 6a, 6d, 7a, 7c.

Sup. Figure 2c – SMAD4 GAPDH

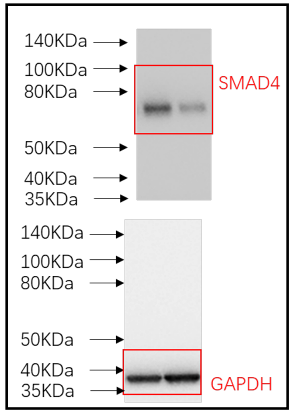

Sup. Figure 2g – SNAIL E-caderin TWIST1 β-actin

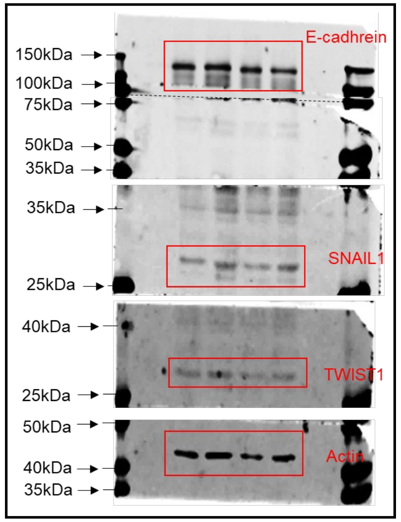

Sup. Figure 3a – PAK3 SMAD4 SMAD3 GAPDH

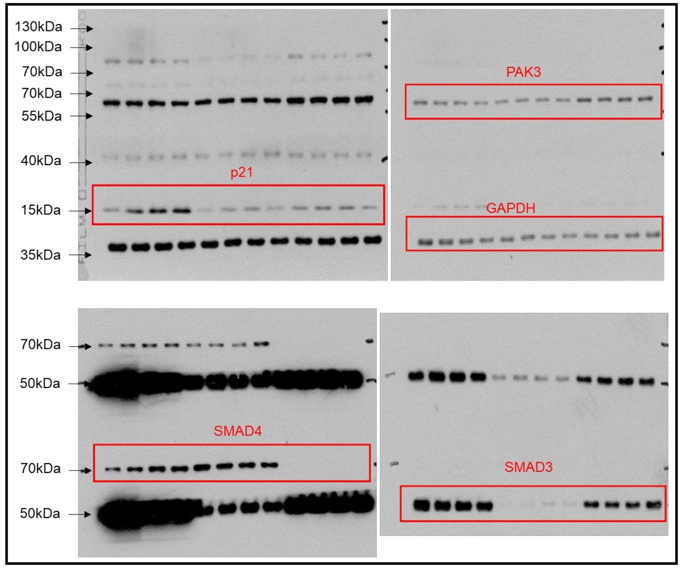

Sup. Figure 4a – PAK3 P-JNK t-JNK P-Jun SMAD4 GAPDH

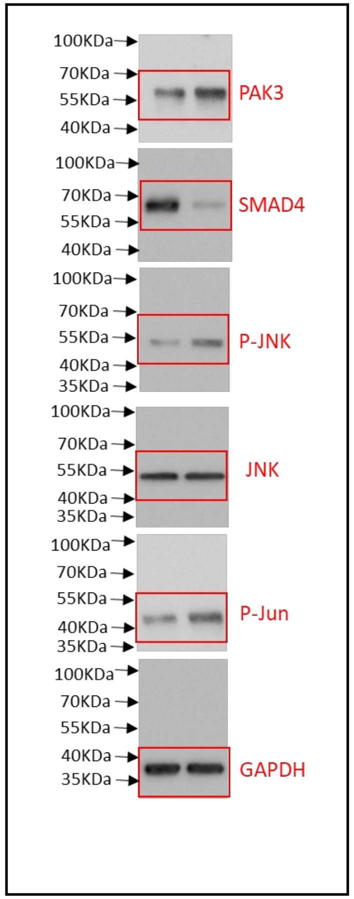

Sup. Figure 4b – PAK3 P-JNK t-JNK P-Jun t-Jun GAPDH

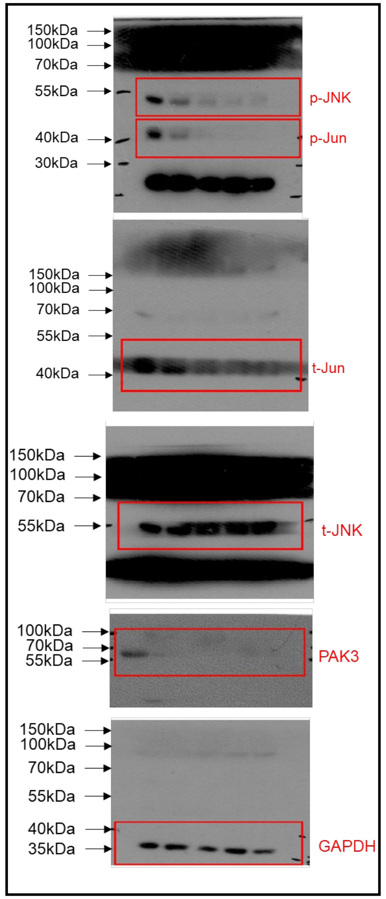

Sup. Figure 4c – Flag P-JNK t-JNK P-Jun t-Jun GAPDH

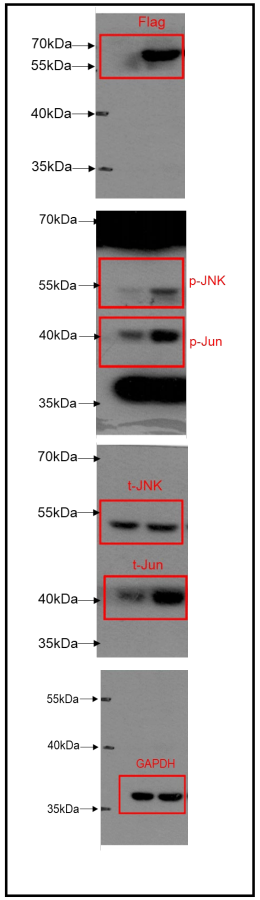

Sup. Figure 4i – P-MEK MEK Actin

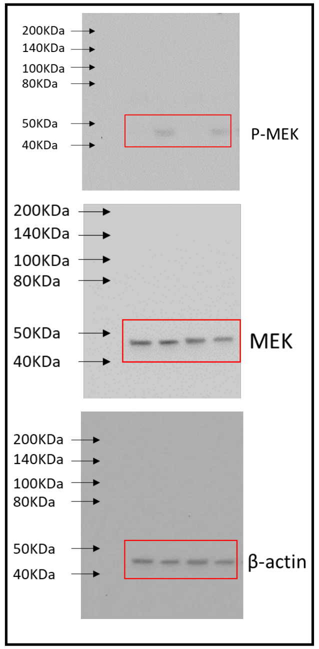

Sup. Figure 5a – SMAD4 β-Actin

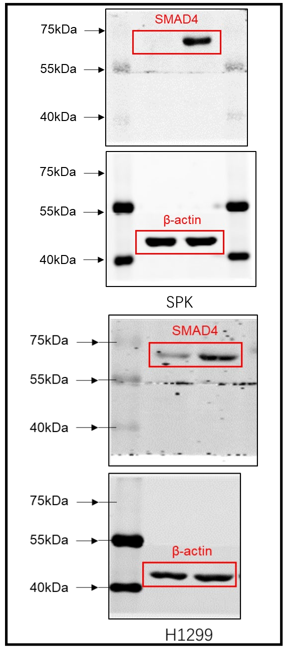

Sup. Figure 5c– SMAD4 β-Actin

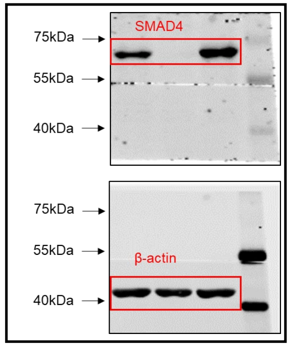

Sup. Figure 6a – SMAD4 β-Actin

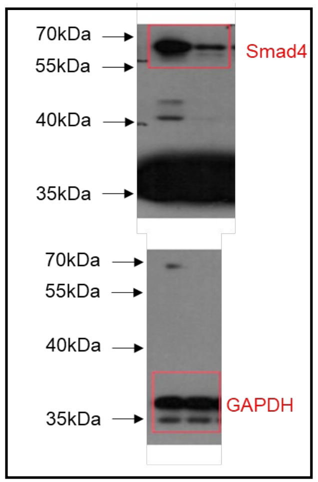

Sup. Figure S6d – SMAD4 GAPDH

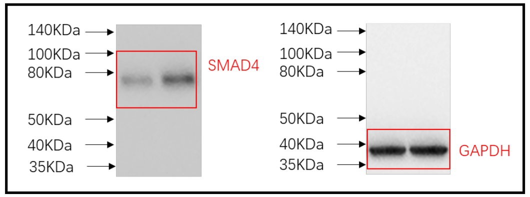

Sup. Figure S7a – SMAD4 β-Actin

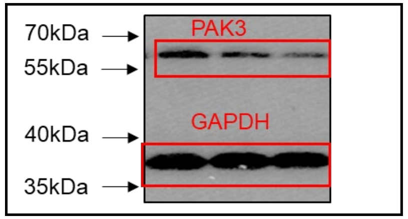

Sup. Figure S7c – PAK3 GAPDH

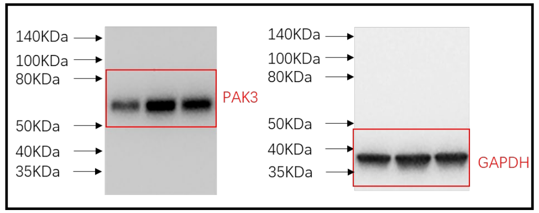

Sup. Table 1 Sequence of primers used in the manuscript.

Genotyping primers

Kras-1: gtctttccccagcacagt; Kras-2: ctcttgcctacgccaccag; Kras-3: agctagccaccatggcttgagt;

p53-1: cgcaatcctttattctgt; p53-2: agcacataggaggcaga; p53-3: tgagacagggtcttgct;

Smad4-1: tgcattccagcctccca; Smad4-2: gccagcagcagcagacagac

RT-PCR primers

mSmad4 sense primer, 5’-AGCCGTCCTTACCCACTGAA-3’; mSmad4 antisense primer, 5’-GGTGGTAGTGCTGTTATGATGGT-3’;

hSmad4 sense primer, 5’-GCTGCTGGAATTGGTGTTGATG-3’; hSmad4 antisense primer, 5’-AGGTGTTTCTTTGATGCTCTGTCT-3’;

mPAK3 sense primer, 5’-CAAAGAAACGGTCAACAACCAG-3’; mPAK3 antisense primer, 5’-AGCTGTTTTGGTATTCGACTGAT-3’;

hPAK3 sense primer, 5’-AGCAATGGGCACGATTACTCC-3’; hPAK3 antisense primer, 5’-GGGCTGCTATGTATCCATGTG-3’;

miR-495 sense primer, 5’-TCGCGCAAACAAACATGGTGCA-3’; miR-495 antisense primer, 5’-CAGTGCAGGGTCCGAGGTAT-3’;

miR-543 sense primer, 5’-TCGCGCAAACAAACATGGTGCA-3’; mi-R-543 antisense primer, 5’-TCGCAAACATTCGCGGTGCA-3’.

18S-F： 5’-GGACACGGACAGGATTGACA-3’; 18S-R： 5’-GACATCTAAGGGCATCACCAG-3’

ChIP qPCR primers

miR-495 Forward primer: 5’- ATGAGAATGGGTTTGGGTTG-3’, miR-495 Reverse primer: 5’- AGCCCTGGGTACTGTCCTCT-3’;

miR-543 Forward primer: 5’- GCAGTAAACAACGCCATCCT-3’, miR-543 Reverse primer: 5’- GATGAGCAAGAACAACGAAGC-3’
