# Supplementary material for: Loss of Smad4 promotes aggressive lung cancer metastasis by de-repression of PAK3 via miRNA regulation
Source: Nat Commun. 2021 Aug 11;12:4853. doi: 10.1038/s41467-021-24898-9 (PMC8357888; doi:10.1038/s41467-021-24898-9)
Supplement: Supplementary file 3 — Description of Additional Supplementary Files [file 41467_2021_24898_MOESM3_ESM.pdf]

Description of Additional Supplementary Files of the manuscript entitled "Loss of Smad4 promotes aggressive lung cancer metastasis by de-repression of PAK3 via miRNA regulation"

File Name: Supplementary Data 1
Description: It includes the differentially expressed genes DEGs between SPK cells and PK cells.

File Name: Supplementary Data 2
Description: It includes the top enriched human diseases by the KEGG analysis of DEGs listed in Supplementary Data 1.

File Name: Supplementary Data 3
Description: It includes the top enriched cellular processes by the KEGG analysis of DEGs listed in Supplementary Data 1.

File Name: Supplementary Data 4
Description: It includes that DEGs, listed in Supplementary Data 1, are related to cancer and cell motility.

File Name: Supplementary Data 5
Description: It is the summary of lung cancer metastasis in PK &SPK mice.

File Name: Supplementary Data 6
Description: It is the summary of SMAD4 mutation in the MSK-IMPACT datasets.

File Name: Supplementary Data 7
Description: It is the summary of lung cancer patients who have triple mutations in KRAS, TP53, and SMAD4.

File Name: Supplementary Data 8
Description: It is the summary of patient information of Figure 7.

File Name: Supplementary Data 9
Description: It is the summary of patient information of Sup. Fig. 9c.
