# Supplementary material for: Loss of Smad4 promotes aggressive lung cancer metastasis by de-repression of PAK3 via miRNA regulation
Source: Nat Commun. 2021 Aug 11;12:4853. doi: 10.1038/s41467-021-24898-9 (PMC8357888; doi:10.1038/s41467-021-24898-9)
Supplement: Supplementary file 13 — Reporting Summary [file 41467_2021_24898_MOESM13_ESM.pdf]

nature research

Corresponding author(s): Xiaotao Li

Last updated by author(s): May 28, 2021

# Reporting Summary

Nature Research wishes to improve the reproducibility of the work that we publish. This form provides structure for consistency and transparency in reporting. For further information on Nature Research policies, see our Editorial Policies and the Editorial Policy Checklist.

## Statistics

For all statistical analyses, confirm that the following items are present in the figure legend, table legend, main text, or Methods section.

| n/a | Confirmed | |
|---|---|---|
| ☐ | ☒ | The exact sample size (*n*) for each experimental group/condition, given as a discrete number and unit of measurement |
| ☐ | ☒ | A statement on whether measurements were taken from distinct samples or whether the same sample was measured repeatedly |
| ☐ | ☒ | The statistical test(s) used AND whether they are one- or two-sided<br>*Only common tests should be described solely by name; describe more complex techniques in the Methods section.* |
| ☒ | ☐ | A description of all covariates tested |
| ☒ | ☐ | A description of any assumptions or corrections, such as tests of normality and adjustment for multiple comparisons |
| ☐ | ☒ | A full description of the statistical parameters including central tendency (e.g. means) or other basic estimates (e.g. regression coefficient) AND variation (e.g. standard deviation) or associated estimates of uncertainty (e.g. confidence intervals) |
| ☐ | ☒ | For null hypothesis testing, the test statistic (e.g. *F*, *t*, *r*) with confidence intervals, effect sizes, degrees of freedom and *P* value noted<br>*Give P values as exact values whenever suitable.* |
| ☒ | ☐ | For Bayesian analysis, information on the choice of priors and Markov chain Monte Carlo settings |
| ☒ | ☐ | For hierarchical and complex designs, identification of the appropriate level for tests and full reporting of outcomes |
| ☐ | ☒ | Estimates of effect sizes (e.g. Cohen's *d*, Pearson's *r*), indicating how they were calculated |

*Our web collection on statistics for biologists contains articles on many of the points above.*

## Software and code

Policy information about availability of computer code

| | |
|---|---|
| Data collection | GraphPad Software (Prism 8); Oncomine analysis;Illumina Genome Analyzer(SCS v2.10); R with various packages; Image-Pro(0.10.6);LI-COR Odyssey Infrared Imaging System (CLx) |
| Data analysis | Prism software (GraphPad Software) was used for statistical analyses. The intensity of the Western blot results was analyzed by densitometry using ImageJ software. Values were shown as mean ± s.e.m. Statistical significance between two samples was determined with two-tailed Student's t-tests. We analyzed the expression of SMAD4 and PAK3 of these patients' tumors and conducted the Oncomine analysis on the published datasets by utilizing the cutoff criteria (P < 0.05; \|log2 Fold change\| > 1.5). The sequencing was performed using Illumina Genome Analyzer. RNA-seq data were analyzed using R with various packages. The differential analysis of genes was conducted on counts using DESeq2 package. Differentially expressed genes (DEGs) were identified as such if the fold change > 2 and the p-value < 0.05. Gene ontology (GO) enrichment and enriched KEGG (Kyoto Encyclopedia of Genes and Genomes) pathways were performed. We utilized Image-Pro to select cells with the positive staining by calculating the grey values. The combination of size ratio between the grey areas and the whole field as well as staining intensity were calculated, and we utilized the resulting index as our definition of the staining intensity (+, +++, etc.). The negative (-) means the percentage of the positive cells with less than 25% and near background staining; the index of the positive cells between 25% and 50% with low intensity is considered as weak (+); those between 50% and 75% with intermediate staining refers to moderate staining (++); those higher than 75% or more than 50% with strongest staining represents intense staining (+++). After incubation with a fluorescent-labelled secondary antibody (Invitrogen; Jackson Immunoresearch, 1:5,000 dilutions), specific signals for proteins were visualized by a LI-COR Odyssey Infrared Imaging System. |

For manuscripts utilizing custom algorithms or software that are central to the research but not yet described in published literature, software must be made available to editors and reviewers. We strongly encourage code deposition in a community repository (e.g. GitHub). See the Nature Research guidelines for submitting code & software for further information.

## Data

Policy information about availability of data

All manuscripts must include a data availability statement. This statement should provide the following information, where applicable:

- Accession codes, unique identifiers, or web links for publicly available datasets
- A list of figures that have associated raw data
- A description of any restrictions on data availability

RNA-Seq data has been deposited into GEO database (GSE164436).We downloaded the raw data from a published paper (PMID: 11707567) using Oncomine website. And then sorted the patients who had lung cancer primary site tumor (n=123) and lung cancer metastasis tumor (n=7) and listed these detailed information in Sup. Data 9.

# Field-specific reporting

Please select the one below that is the best fit for your research. If you are not sure, read the appropriate sections before making your selection.

☒ Life sciences ☐ Behavioural & social sciences ☐ Ecological, evolutionary & environmental sciences

For a reference copy of the document with all sections, see nature.com/documents/nr-reporting-summary-flat.pdf

# Life sciences study design

All studies must disclose on these points even when the disclosure is negative.

| | |
|---|---|
| Sample size | Group size was determined based on the results of preliminary experiments (PMID: 31089135), especially depending on the tumor incidence of each mouse line and no statistical method was used to predetermine sample size in animal studies. For each animal experiments, the number, such n = 6, has been indicated with each figure in the manuscript. |
| Data exclusions | No data was excluded. |
| Replication | All the results haven been biologically and successfully repeated three times or more. |
| Randomization | Yes, all the animal were randomly to be grouped in all the experiments. To implement random assignment, we assigned a unique number to every member of our study's sample. Then, we used a random number generator to randomly assign each number to a control or experimental group. |
| Blinding | All the bioinformatic and statistical analyses were unbiasedly analyzed or confirmed by bioinformaticians. The genotyping of mice was confirmed independently by two students. All the array experiments were unbiasedly conducted and analyzed by the sequencing company. All qRT-PCR experiments were performed in a blinded manner. The investigators were blinded to group allocation during data collection and/or analysis for bioinformatic and statistical analyses and array experiments.<br>The routine experiments, such as collecting the cells or mouse tissues, or preparing the buffer, need clearly recorded information. It is important for the whole lab. Therefore, it is not feasible to perform these routine experiments in a blinded manner. |

# Reporting for specific materials, systems and methods

We require information from authors about some types of materials, experimental systems and methods used in many studies. Here, indicate whether each material, system or method listed is relevant to your study. If you are not sure if a list item applies to your research, read the appropriate section before selecting a response.

### Materials & experimental systems

| n/a | Involved in the study | |
|---|---|---|
| ☐ | ☒ | Antibodies |
| ☐ | ☒ | Eukaryotic cell lines |
| ☒ | ☐ | Palaeontology and archaeology |
| ☐ | ☒ | Animals and other organisms |
| ☐ | ☒ | Human research participants |
| ☒ | ☐ | Clinical data |
| ☒ | ☐ | Dual use research of concern |

### Methods

| n/a | Involved in the study | |
|---|---|---|
| ☒ | ☐ | ChIP-seq |
| ☒ | ☐ | Flow cytometry |
| ☒ | ☐ | MRI-based neuroimaging |

## Antibodies

| | |
|---|---|
| Antibodies used | Smad4 (Santa Cruz, sc-7966, clone: B-8, lot: H0212, 1:200), βPAK (Santa Cruz, sc-1871, clone: N-19, lot: J1512, 1:100), p-c-Jun (CST #2361, clone: 54B3, lot: 7, 1:100), p-JNK (CST #4668, clone: 81E11, lot: 11, 1:50), Ras (G12D) Mutant (CST #14429, clone: D8H7, lot: 1, 1:50), P53 (Santa Cruz sc-126, clone: DO-1, lot: L5479, 1:50), TTF1 (Abcam ab76013, clone: EP1584Y, lot: GR297063-7, 1:200).PAK |

(Santa Cruz, sc-1871, clone: N-19, lot: J1512, 1:200), PAK3 (CST #2609T, Clonality: Polyclonal, lot:2, 1:1000), c-Jun (CST #9165S, clone: 60A8, lot: 11, 1:1000), p-c-Jun (CST #2361, clone: 54B3, lot: 7, 1:1000), JNK (CST #9258S, clone: 56G8, lot: 11, 1:1000), p-JNK (CST #4668, clone: 81E11, lot: 11, 1:1000), Smad4 (CST #46535, clone: D3R4N, lot:2, 1:1000), GAPDH (Proteintech 60004-1-IG, clone: 1E6D9, lot: 10013030, 1:1000), p-MEK1 (Ser298) (Abcam, ab96379, clone: EPR3338, Lot: GR251630-1), MEK1 (Abcam, ab32576, clone: Y77, Lot: GR245617-1), E-Cadherin (CST, #3195, clone: 24E10, Lot:13), TWIST1 (CST, #69366, clone: E7E2G, Lot:1), SNAIL1 (Abcam, ab216347, clone: EPR21043, Lot: GR216730-1), β-actin (MBL M177-3, clone: 6D1, lot: 008, 1:5000).Alexa Fluor® 680 AffiniPure Goat Anti-Mouse IgG (H+L), Jackson ImmunoResearch, Code: 115-625-146, Clonality: Polyclonal, lot: 146048, RRID: AB_2338935. Alexa Fluor® 790 AffiniPure Goat Anti-Mouse IgG (H+L), Jackson ImmunoResearch, Code: 115-655-146, Clonality: Polyclonal, lot: 108636, RRID: AB_2338944. Alexa Fluor® 790 AffiniPure Goat Anti-Rabbit IgG (H+L), Jackson ImmunoResearch, Code: 111-655-144, Clonality: Polyclonal, lot: 134979, RRID: AB_2338086.

Validation

All the antibodies can be validated in the published studies, listed by PMID numbers.

Smad4 (Santa Cruz, sc-7966, clone: B-8, lot: H0212, 1:200)(PMID: # 33990575), βPAK (Santa Cruz, sc-1871, clone: N-19, lot: J1512, 1:100) (PMID: 31943058), p-c-Jun (CST #2361, clone: 54B3, lot: 7, 1:100)(PMID: 32877278), p-JNK (CST #4668, clone: 81E11, lot: 11, 1:50) (PMID: 33717117), Ras (G12D) Mutant (CST #14429, clone: D8H7, lot: 1, 1:50)(PMID: 33087508), P53 (Santa Cruz sc-126, clone: DO-1, lot: L5479, 1:50)(PMID: # 33491667), TTF1 (Abcam ab76013, clone: EP1584Y, lot: GR297063-7, 1:200)(PMID: 32005814).PAK3 (CST #2609T, Clonality: Polyclonal, lot:2, 1:1000)(PMID: 32320648), c-Jun (CST #9165S, clone: 60A8, lot: 11, 1:1000)(PMID: 33627422), p-c-Jun (CST #2361, clone: 54B3, lot: 7, 1:1000)(PMID: 32877278), JNK (CST #9258S, clone: 56G8, lot: 11, 1:1000)(PMID: 33330641), p-JNK (CST #4668, clone: 81E11, lot: 11, 1:1000)(PMID: 33665189), Smad4 (CST #46535, clone: D3R4N, lot:2, 1:1000) (PMID: 33179861), GAPDH (Proteintech 60004-1-IG, clone: 1E6D9, lot: 10013030, 1:1000)(PMID: 28701303), p-MEK1 (Ser298) (Abcam, ab96379, clone: EPR3338, Lot: GR251630-1)(PMID: 32567007), MEK1 (Abcam, ab32576, clone: Y77, Lot: GR245617-1) (PMID: 30876463), E-Cadherin (CST, #3195, clone: 24E10, Lot:13)(PMID: 33637728), TWIST1 (CST, #69366, clone: E7E2G, Lot:1) (PMID: 32945479), SNAIL1 (Abcam, ab216347, clone: EPR21043, Lot: GR216730-1)(PMID: 33692334), β-actin (MBL M177-3, clone: 6D1, lot: 008, 1:5000)(PMID: 29773794). Alexa Fluor® 680 AffiniPure Goat Anti-Mouse IgG (H+L), Jackson ImmunoResearch, Code: 115-625-146, Clonality: Polyclonal, lot: 146048, RRID: AB_2338935 (PMID: 29216499). Alexa Fluor® 790 AffiniPure Goat Anti-Mouse IgG (H+L), Jackson ImmunoResearch, Code: 115-655-146, Clonality: Polyclonal, lot: 108636, RRID: AB_2338944 (PMID: 29605410). Alexa Fluor® 790 AffiniPure Goat Anti-Rabbit IgG (H+L), Jackson ImmunoResearch, Code: 111-655-144, Clonality: Polyclonal, lot: 134979, RRID: AB_2338086 (PMID: 32359423).

# Eukaryotic cell lines

Policy information about cell lines

Cell line source(s)

mouse lung cancer derived cell lines PK and SPK; H1299 cells were from ATCC.

Authentication

The mouse lung cancer derived cell lines PK and SPK were established from in vivo PK and SPK tumors. Briefly, tumors were dissected 12 weeks post Ad-Cre virus treatment, minced into small pieces and digested with collagenase for 1 hour at 37°C. Digested tissue were filtered through a 100 um filter, then a 40 um filter using excess cold PBS to wash cell through filter. Finally, the tumor cells were cultured in RPMI-1640 (Hyclone) with 10% FBS. The medium was changed every day until cells outgrew and stable immortalized cell lines were formed. H1299 cells were from ATCC. The STR profiling of our H1299 cells is "
Amelogenin:X
CSF1PO:12
D13S317:12
D16S539:12,13
D5S818:11
D7S820:10
TH01:6,9.3
TPOX:8
vWA: 16,17,18".

Mycoplasma contamination

These cells are not examined for mycoplasma.

Commonly misidentified lines
(See ICLAC register)

PK cells are the cells with the P53 deletion and KrasG12D mutation. SPK cells are the cells with the P53 deletion and KrasG12D mutation, as well as SMAD4 deletion. No commonly misidentified cell lines were used in the study.

# Animals and other organisms

Policy information about studies involving animals; ARRIVE guidelines recommended for reporting animal research

Laboratory animals

All strains were B6. 129 and described in detail in the Method section. Please find the mouse information in the Sup. Table 1 and Figure 1C. The mouse housing conditions are "a 14-hour light/10-hour dark cycle and temperatures of 65-75°F (~18-23°C) with 40-60% humidity".

Wild animals

No wild animals were used in the study.

Field-collected samples

No field collected samples were used in the study.

Ethics oversight

All experiments were conducted under specific pathogen free (SPF) conditions and handled according to the ethical and scientific standards by the Animal Center at Shanghai Key Laboratory of Regulatory Biology, Institute of Biomedical Sciences, School of Life Sciences, East China Normal University following procedures approved by the Institutional Animal Care and Use Committee.

Note that full information on the approval of the study protocol must also be provided in the manuscript.

## Human research participants

Policy information about studies involving human research participants

| | |
|---|---|
| Population characteristics | Please find the population characteristics in the Sup. Table 4. |
| Recruitment | Provide written information to patients. Educate patients about the related researches and trials before asking for their consent. Ensure that the patients are well informed about the researches and trials. The collected samples were provided by the doctors without any bias selections. |
| Ethics oversight | Human specimens used in this study have been approved by the East China Normal University. |

Note that full information on the approval of the study protocol must also be provided in the manuscript.
